# Port calls and vessel trajectory dataset in the Caribbean with accurate port quays survey

**DOI:** 10.1016/j.dib.2024.110617

**Published:** 2024-06-10

**Authors:** Clément Iphar, Iwan Le Berre, Manuel Sahuquet, Aldo Napoli, Éric Foulquier

**Affiliations:** a LETG-Brest GEOMER, UMR 6554 CNRS, IUEM-Université de Bretagne Occidentale, Rue Dumont D’Urville, F-29280 Plouzané, France; b CRC, Mines Paris - PSL, Rue Claude Daunesse, Sophia Antipolis, France

**Keywords:** Port calls, Automatic identification system, Vessels, Port infrastructures, Maritime transport, Statistics

## Abstract

With the growth in maritime traffic comes an increased need for precise modelling, analysis, and visualisation to enhance the monitoring capabilities of maritime authorities. To address this need, a range of sensing technologies have been developed to track vessel movements worldwide. Among these, the Automatic Identification System (AIS) is particularly significant, offering high-frequency transmission of both location and identification data. This makes AIS an invaluable tool in the intricate process of modelling maritime traffic that we use in this study. Our study presents a comprehensive dataset for the Caribbean in 2019, including port calls, quay geometries, vessel trajectories, daily locations, a seven-class vessel classification, port statistics, and United Nations reference data for comparison. Beneficial for geomatics, geography, and economics, the dataset provides a versatile tool for visualising data, assessing maritime impact on coastal areas, and enhancing maritime trade analysis. The methodology extracts 1.5 million port calls from 642 million AIS messages, offering detailed data tables and reusable processes. Its granularity down to the single quay allows for flexible data analysis, facilitating in-depth understanding of port and inter-port maritime activities.

Specifications Table

**Table:** 

Subject	Maritime Mobility and Transport
Specific subject area	Determination of port calls, Computation of characteristics of port calls and of the corresponding vessels, Determination of vessel trajectories
Type of data	Text data files, data is stored in CSV, and additionally, for the tables that have a geographical field, in geojson and in shapefile formats
Data collection	Port quays have been retrieved by GIS photo-interpretation for the 528 ports of the area of interest. Port calls have been computed from a set of AIS data contacts. Berths have been computed from ports and port calls. Area presence has been computed from raw AIS data, Trajectories have been computed from port calls. Vessel types have been computed from IHS Markit dataset [2] and berths. Segments have been computed from trajectories. Port traffic has been computed from port calls and IHS Markit dataset. UNCTAD tables have been retrieved from reference data extracted from UNCTAD website [3].
Data source location	Caribbean Sea and islands, Gulf of Mexico, Eastern Florida shore, Bahamas, Guianas
Data accessibility	Zenodo deposit [4], named “Port calls and vessel trajectory dataset in the Caribbean with accurate port quays survey” Usage rights: Creative Commons Attribution-NonCommercial-ShareAlike 4.0 International (CC BY-NC-SA 4.0) or Creative Commons Attribution 3.0 Intergovernmental Organisations (CC BY 3.0 IGO) Repository name: Zenodo Data identification number: doi:10.5281/zenodo.10091946 - Version 1.1.0, doi of version: 10.5281/zenodo.10380638 Direct URL to data: https://zenodo.org/records/10380638
Related research article	C. Iphar, I. Le Berre, A. Napoli, É. Foulquier, Port Call Extraction from Maritime Navigation Data for Port Activity Estimation, Ocean Engineering 293 (2024). https://doi.org/10.1016/j.oceaneng.2024.116771

## 1 Value of the Data

• The port call and vessel trajectory dataset with accurate quay survey provide at once (1) a set of port calls for the Caribbean area for the whole of 2019, together with (2) the geometry of quays and wharves of the said ports, (3) the trajectories and subsequent segments, (4) the location of vessels on a daily basis, (5) a classification of vessels in seven classes, (6) a set of port statistics, (7) geometries differentiated by dock type and (8) reference data from the United Nations Conference for Trade and Development to compare against.
• Research in the fields of geomatics, geography or economy can benefit from this paper. In geomatics for the possibilities of data visualisation from a variety of parameters, in geography to assess the impact of maritime traffic on the coastal territories and population, including exposure to pollution, and in economy to offer a differentiated approach and detailed information about maritime trade.
• The general method for the computation of all data tables is presented in detail in this paper. It enables turning a raw AIS dataset of 642 million messages into a set of 1.5 million port calls. A clear and precise description of all data tables and processes leading to their generation is provided, allowing reusability.
• The great detail in both the differentiated approach by vessel type and the precise computation of the location of the port call, in conjunction with the accurate survey of all commercial quays and wharves of the Caribbean, offer a great degree of granularity, as the user is not limited to the predefined 528 ports, but can group them or divide them as one wishes. This freedom of choice in the granularity level of data offers perspectives in many fields and provides a support for understanding maritime activities at port, and between ports.
• The generated dataset of eleven tables is completed with three tables that are excerpts of official United Nations Conferences on Trade and Development, giving country-aggregated data on port calls, and goods throughput, providing both reference data for, on the one hand any side-product from this dataset and, on the other hand for the validation of the method, as shown in [5].

## 2 Background

This dataset stems from the needs of Human-Environment Observatory for the Caribbean Coast (OHM Littoral Caraïbe) to better understand the dynamics of marine traffic around the archipelago of Guadeloupe, and at a larger scale in the whole of the Caribbean. The Observatory pursues goals such as modelling the socio-economical stakes and environmental pressures of maritime traffic, both at sea and on the coastal populations. In this respect, the extraction of port calls and other topical information is of great added value, enabling to assess the location, duration, and nature of such port calls. This data paper offers a description of the data, thus enhancing its accessibility and potential of reuse, that has been generated and validated through the method presented in [5].

## 3 Data Description

The dataset is composed of fourteen data files: the first eleven being the handcrafted and computed tables and the last three being the reference tables. Table 1 presents some characteristics of the fourteen data files. The sizes shown in Table 1 are for CSV files. The total size of CSV files is 289 Mb, plus 498 Mb for geojson files and 48 Mb for shapefiles, amounting to an all-encompassing total of 835 Mb.

**Table 1:** List of the data files of the published dataset.

File	File name	Size	# rows	Type	Sep.	SRID	Licence
**Port calls**	Portcalls	202 Mb	1,488,781	CSV, SHP, geojson	|	4326	CC-BY-NC-SA-4.0
**Port quays and wharves**	Ports	459 Kb	528	CSV, SHP, geojson	|	4326	CC-BY-NC-SA-4.0
**Berths**	Berths	516 Kb	1,902	CSV, SHP, geojson	|	4326	CC-BY-NC-SA-4.0
**Trajectories**	Trajectories	74 Mb	1,473,752	CSV	|	N/A	CC-BY-NC-SA-4.0
**Segments between ports**	segments_port2port	1.2 Mb	9,969	CSV, SHP, geojson	|	4326	CC-BY-NC-SA-4.0
**Segments between ports and territories**	segments_port2territory	895 Kb	7,425	CSV, SHP, geojson	|	4326	CC-BY-NC-SA-4.0
**Vessel types from registry**	vesseltype_original	258 Kb	12,476	CSV	|	N/A	CC-BY-NC-SA-4.0
**Vessel type - enriched**	vesseltype_enriched	70 Kb	2,643	CSV	|	N/A	CC-BY-NC-SA-4.0
**Vessel area location**	Areapresence	16 Mb	35,652	CSV	|	N/A	CC-BY-NC-SA-4.0
**Docking type**	Docking	773 Kb	3	CSV, SHP, geojson	|	4326	CC-BY-NC-SA-4.0
**Port traffic statistics**	port_traffic	66 Kb	528	CSV	|	N/A	CC-BY-NC-SA-4.0
**UNCTAD port calls by country**	ungt_country_portcalls	2 Kb	38	CSV	|	N/A	CC BY 3.0 IGO
**UNCTAD port call statistics by country**	ungt_country_portstats	18 Kb	333	CSV	|	N/A	CC BY 3.0 IGO
**UNCTAD container throughput**	ungt_cont_throughput	1 Kb	25	CSV	|	N/A	CC BY 3.0 IGO

In the remainder of this section, each file will be presented and each data feature, corresponding to the columns in the files, are shown in each line of Table 2, Table 3, Table 4, Table 5, Table 6, Table 7, Table 8, Table 9, Table 10, Table 11, Table 12, Table 13, Table 14, with a precision on their nature, their universe of discourse and a short description.

**Table 2:** Description of the data features of the “Port calls” data file.

Feature	Type	Universe of discourse	Short description
**id**	Integer	[1;1,488,781]	Point identifier, primary key
**mmsi**	Integer	[45;1,073,709,206]	Unique vessel identifier number, as defined by the IMO and assigned by countries
**port**	Integer	[1;528]	Unique identifier of port number. Foreign key to the primary key of the ports table
**ts_start**	Timestamp	[2019-01-01 00:00:00 - 2019-12-31 23:59:59]	Beginning of the port call
**ts_end**	Timestamp	[2019-01-01 00:00:00 - 2019-12-31 23:59:59]	End of the port call
**nb_mes**	Integer	N^+^*	Total number of received messages between the first and the last messages of the port call (not included)
**time_bef**	Integer	N^+^	(if applicable) Timeframe in seconds between the beginning of the port call and the last message received prior to it
**time_aft**	Integer	N^+^	(if applicable) Timeframe in seconds between the end of the port call and the first message received after it
**duration**	Integer	N^+^	In seconds, time elapsed between ts_start and ts_end
**conf**	Real	[0;1]	Degree to which the computed duration is deemed credible of being the actual duration
**latitude**	Real	]-90;90]	(if applicable) mean latitude of the vessel while stopped during the port call
**longitude**	Real	]-180;180]	(if applicable) mean longitude of the vessel while stopped during the port call
**geom**	geometry		(if applicable) geometry point of latitude and longitude values

### 3.1 Port calls file

**Fig. 1:**
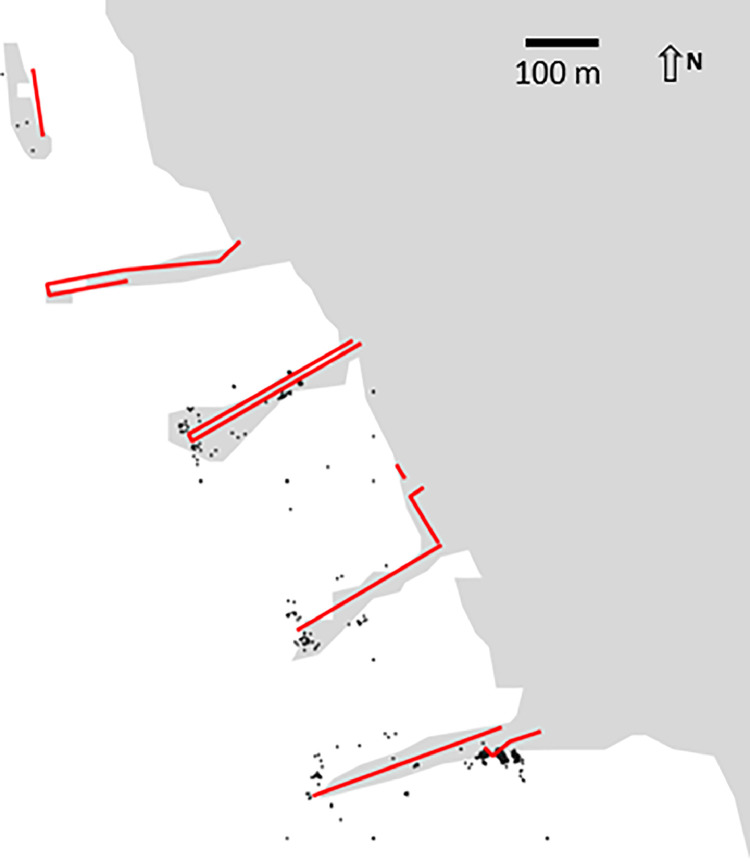
location of port calls (black dots) around quays and wharves (red lines) in the Venezuelan port of Punta Cardón.

This file lists all the port calls computed in the Caribbean for the year 2019. Each of the port calls is defined by an entry. The feature **id** constitutes the primary key of the table and once the points have been sorted by ascending value of their primary key. The total number of calls is 1,488,781, unevenly distributed across ports and inside ports as shown later in Fig. 1 with the example of the Venezuelan port of Punta Cardón. Table 2 details the characteristics of the fields of this file, which is named portcalls.csv.

### 3.2 Port quays and wharves file

**Fig. 2:**
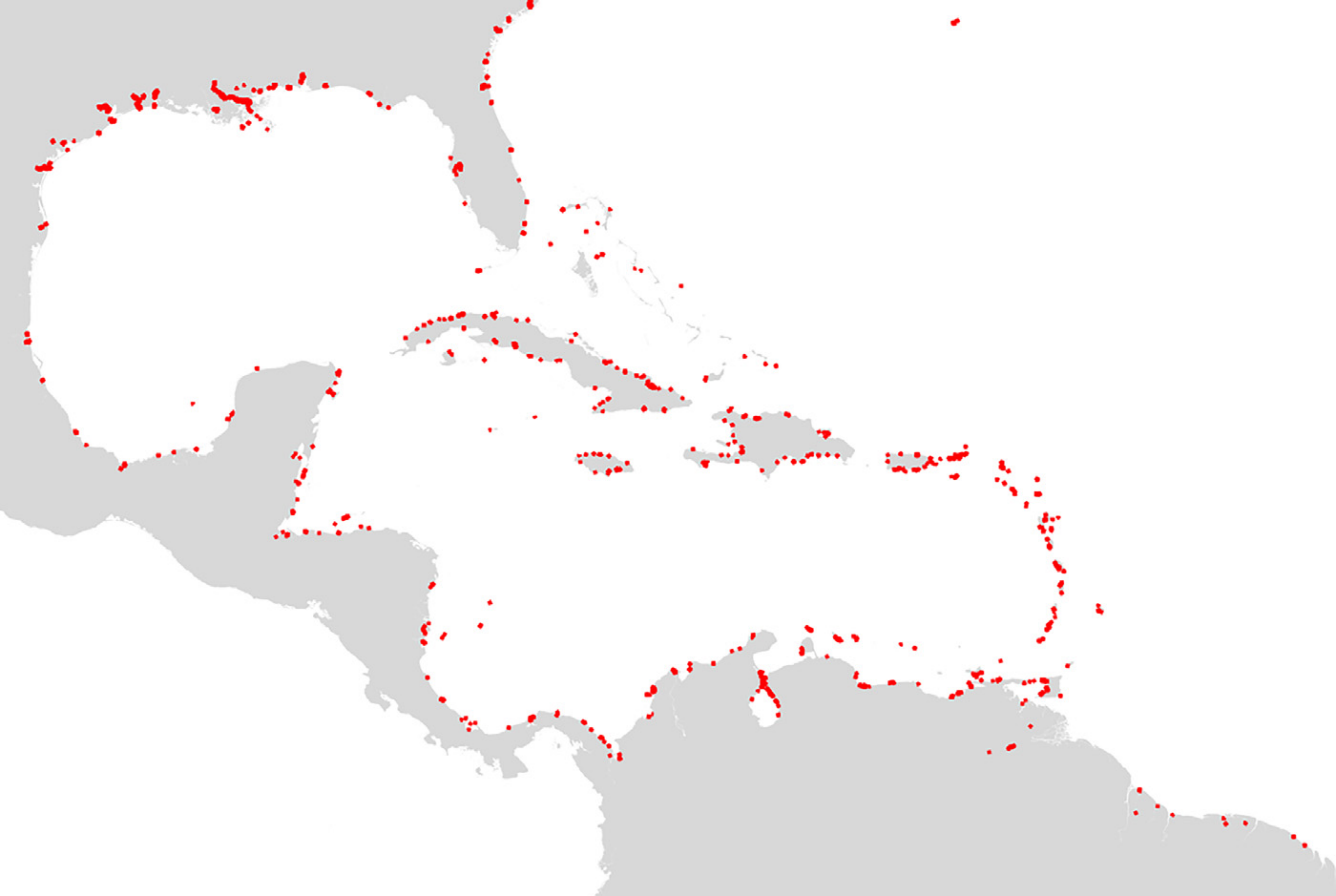
Location of all 528 ports of the area of interest.

This file lists all 528 commercial port sites of the area of interest, and more particularly displays in great detail the quays and the wharves of each port. This table has been manually generated by the authors using GIS photo-interpretation. In order to provide an overview of all port infrastructures capable of receiving merchant vessels, digitalisation was carried out at a scale of 1:5000, using *Google Satellite, Google Earth* and *Bing Map* satellite imagery, and the *OpenStreetMap* cartographic repository. A total of 528 commercial port sites, spanning across 41 territories (sovereign nations or dependencies) have been identified and labelled. They are shown in Fig. 2. The feature id constitutes the primary key of the table and is, throughout this document and the dataset, the identifier of ports. Table 3 details the characteristics of the fields of this file, which is named ports.csv.

**Table 3:** Description of the data features of the “ports” data file.

Feature	Type	Universe of discourse	Short description
**Id**	Integer	[1;528]	Port identifier, primary key
**portname**	Text	{List of ports}	Name of the port (usually the name of the city, not the commercial name of the port or port authority)
**countryname**	Text	{List of country names}	Name of the country within which the port is located
**countrycode**	Text	{List of country codes}	3-letter code of the country, following ISO 3166-1 alpha-3 norm
**quay**	Integer	[0;59,384]	(if applicable) Length in meters of all the quays of the port
**wharf**	Integer	[0;79,468]	(if applicable) Length in meters of all the wharves of the port
**offshore**	Integer	[0;832]	(if applicable) Length in meters of all the offshore landing stages of the port
**geom**	Geometry		MultiLineString geometry of all quay, wharf and offshore features of the port
**locode**	Text	{List of codes}	(if applicable) official LOCODE of the port, following UNECE nomenclature

### 3.3 Berths file

The file entitled berths.csv lists all areas, called berths, in which a cluster of positions of vessels has been recorded during their port call, corresponding to the field *geometry* of Table 2. Those clusters have been surveyed manually and identified in a unique manner following the nomenclature XX_YY, where XX stands for the identifying number of the port, corresponding to the field *id* of Table 3, and YY is an increment, starting at 01 and going up at each new berth for each given port. A total of 1,902 of such berths have been identified across the 528 ports. Table 4 details the characteristics of the fields of this file.

**Table 4:** Description of the data features of the “berths” data file.

Feature	Type	Universe of discourse	Short description
**id**	Integer	[1;528]	Port identifier, primary key
**portname**	Text	{List of ports}	Name of the port, same as in Table 3
**portid**	Integer	[1;528]	Number of the port, foreign key to the primary key of the ports table (Table 3)
**countrycode**	Text	{List of country codes}	3-letter code of the country, following ISO 3166-1 alpha-3 norm
**berth**	Text	XX_YY	Code of the berth
**number**	Integer	[0;33,888]	Number of calls in the given berth
**inport**	Real	[0;1]	Proportion of the number of calls in the berth with respect to all the calls at the given port
**geom**	Geometry		Polygon geometry of the berth

### 3.4 Vessel trajectories file

This file, named trajectories.csv, aims at recording all the successive port calls for vessels under the form of semantic trajectories. The two columns port_origin and port_destination show each movement of each vessel between ports, including when successive port calls occur in the same port. The purpose of trajectories is also to consider entries and exits from the area of interest, in this respect, three entry/exit areas have been identified, corresponding to the three areas presented later in Section 3.9: (a) towards or from the North Atlantic area, (b) towards or from the South Atlantic area, (c) through the Panama Canal. In order to integrate these areas into the chain of ports visited by a ship, three identifiers have been assigned to these areas. They serve as the start and end points of the semantic trajectories if the ship is outside the Caribbean zone at any given time during the year. This ensures that the semantic trajectories are not distorted by ignoring the fact that the vessel has left the zone. Those areas, for inclusion in the semantic trajectories, have been assigned numbers, which are 997 for the North Atlantic, 998 for the South Atlantic and 999 for the Panama Canal. Successive port calls are gathered in trajectories, and the whole file is ordered as follows: first by MMSI number, then by trajectory, then by the position of the segment in the given trajectory. Table 5 details the characteristics of the fields of this file.

**Table 5:** Description of the data features of the “trajectories” data file.

Feature	Type	Universe of discourse	Short description
**id**	Integer	[1;1,473,752]	Identifier, primary key
**idtraj**	Integer	[1;83]	Rank of the trajectory of the year for the given mmsi, determined incrementally
**rankintraj**	Integer	[1;4374]	Rank of the segment of the corresponding trajectory number **idtraj** of the year for the given mmsi
**mmsi**	Integer	[45;1,073,709,206]	Unique vessel identifier number, as defined by the IMO and assigned by countries
**port_origin**	Integer	[1;528]∪{997,998,999}	Code of the origin of the segment, in [1;528] if it is a port (references **id** column of the “ports” file) or in {997,998,999} if it is a entry/exit area
**port_destination**	Integer	[1;528]∪{997,998,999}	Code of the destination of the segment, in [1;528] if it is a port (references **id** column of the “ports” file) or in {997,998,999} if it is a entry/exit area
**ts**	Timestamp	[2019-01-01 00:00:00 - 2019-12-31 23:59:59]	Date of beginning of the jth segment of the ith trajectory of the given mmsi

### 3.5 Network segments between ports file

This file, named segments_port2port.csv, aggregates all the segments shown in the trajectories file presented in Section 3.4, in order to determine the cardinality of each individual segment. The segments are considered to be directional. Therefore if port A and port B see traffic occur between them in both directions two individual entries will be generated. Table 6 details the characteristics of the fields of this file.

**Table 6:** Description of the data features of the “segments_port2port” data file.

Feature	Type	Universe of discourse	Short description
**id**	Integer	[1;9,969]	Identifier, primary key
**port_origin**	Integer	[1;528]	The identifier of the port of origin, references **id** column of the “ports” file
**port_destination**	Integer	[1;528]	The identifier of the port of destination, references **id** column of the “ports” file
**number_all**	Integer	N+*	Number of travels any vessel did from port_origin to port_destination
**number_bulk**	Integer	N+	Number of travels a bulk vessel did from port_origin to port_destination
**number_cont**	Integer	N+	Number of travels a container vessel did from port_origin to port_destination
**number_crui**	Integer	N+	Number of travels a cruise vessel did from port_origin to port_destination
**number_gcar**	Integer	N+	Number of travels a general cargo vessel did from port_origin to port_destination
**number_iisl**	Integer	N+	Number of travels an inter-island vessel did from port_origin to port_destination
**number_serv**	Integer	N+	Number of travels a service vessel did from port_origin to port_destination
**number_tank**	Integer	N+	Number of travels a tanker vessel did from port_origin to port_destination
**geom**	geometry		LineString geometry linking port_origin centre of mass to port_destination centre of mass

### 3.6 Network segments between ports and territories file

**Fig. 3:**
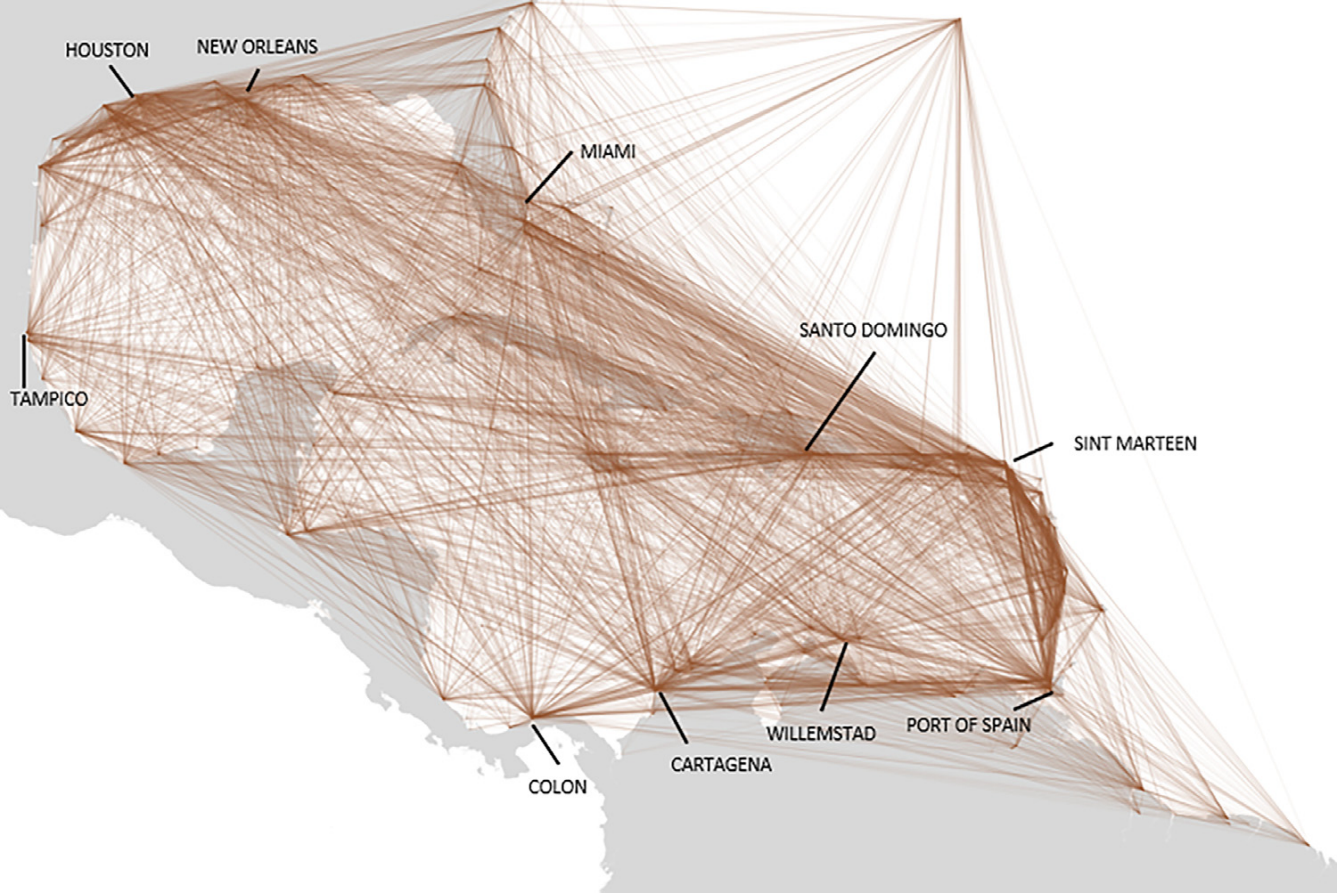
A representation of port to port segments.

This file, named segments_port2territory.csv, aggregates all the segments from the segments_port2port file presented in Section 3.5, by country (of origin or of destination of the segment). Therefore, for each port, each country (or territorial dependency) with at least one segment which is an origin or a destination for the said port will have an entry in the table. Each segment is then composed of a pair port/country. To specify the direction of the segment, the direction field indicates 1 if the segment originates from the port, or 0 if its destination is the port. Fig. 3 shows all the segments in an all-encompassing view of the port to port relations. Table 7 details the characteristics of the fields of this file.

**Table 7:** Description of the data features of the “segments_port2territory” data file.

Feature	Type	Universe of discourse	Short description
**Id**	Integer	[1;7,425]	Identifier, primary key
**port**	Integer	[1;528]	The identifier of the port involved in the segment, references **id** column of the “ports” file
**country**	Text	{List of country codes}	3-letter code of the territory involved in the segment, references the column **countrycode** of the “ports” file
**direction**	Integer	{0,1}	Direction of the segment. Port to country if value is 1, country to port if the value is 0
**number_all**	Integer	N+*	Number of travels any vessel did between the said port and country
**number_bulk**	Integer	N+	Number of travels a bulk vessel did between the said port and country
**number_cont**	Integer	N+	Number of travels a container vessel did between the said port and country
**number_crui**	Integer	N+	Number of travels a cruise vessel did between the said port and country
**number_gcar**	Integer	N+	Number of travels a general cargo vessel did between the said port and country
**number_iisl**	Integer	N+	Number of travels an inter-island vessel did between the said port and country
**number_serv**	Integer	N+	Number of travels a service vessel did between the said port and country
**number_tank**	Integer	N+	Number of travels a tanker vessel did between the said port and country
**geom**	geometry		LineString geometry linking port centre of mass and a point in the considered country

### 3.7 Extracted vessel types file

This file, named vesseltype_original.csv, gathers all the vessels for which the type is known and has been extracted from a database that we acquired from IHS Markit. This file is of critical importance in order to properly assign vessels to their corresponding classes and therefore have a differentiated approach to maritime traffic. Table 8 details the characteristics of the fields of this file.

**Table 8:** Description of the data features of the “vesseltype_original” data file.

Feature	Type	Universe of discourse	Short description
**id**	Integer	[1;12,476]	Identifier, primary key
**mmsi**	Integer	[205,146,000;775,995,140]	Unique vessel identifier number, as defined by the IMO and assigned by countries
**vesseltype**	Text	{bulk,cont,crui,gcar,iisl,serv,tank}	Type of vessel

### 3.8 Inferred vessel types file

This file, named vesseltype_enriched.csv, gathers all the vessels for which the type is not known from the purchased IHS Markit fleet register, but rather inferred from our port calls and berths. Since similar vessels tend to stop at similar locations, if all or nearly all port calls registered in a place are from one specific vessel type, provided that the share of known calls is important enough, it is reasonable to assume the type of the other vessel calling at the exact same quay or wharf. Only cases for which the share of known calls belonging to the same type is over 0.9 are kept in this table, so that the certainty of this inferred data is high. Table 9 details the characteristics of the fields of this file.

**Table 9:** Description of the data features of the “vesseltype_enriched” data file.

Feature	Type	Universe of discourse	Short description
**id**	Integer	[1;12,476]	Identifier, primary key
**mmsi**	Integer	[205,146,000;775,995,140]	Unique vessel identifier number, as defined by the IMO and assigned by countries
**vesseltype**	Text	{bulk,cont,crui,gcar,iisl,serv, tank}	Type of vessel
**share**	Real	[0,1]	Share of calls from vessels in the corresponding berths that had this vessel type

### 3.9 Vessel visibility file

**Fig. 4:**
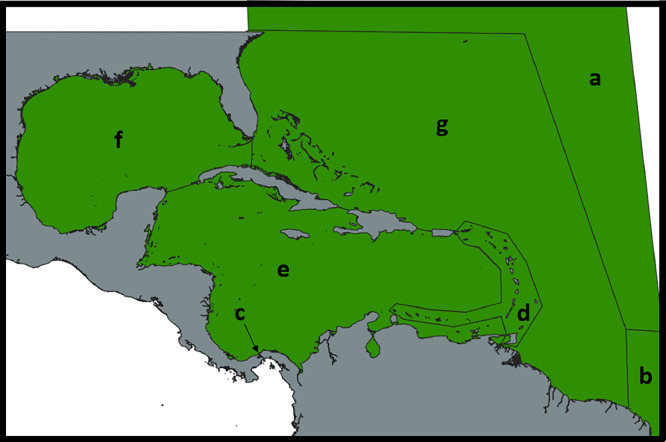
the partition of the space in seven areas of interest.

This file, named areapresence.csv, describes the geographical location of all vessels on a daily basis. The whole geographical area of interest has been divided into 7 zones, of which 3 are specifically zones of entry/exit, and 4 are zones in which ports of interest are located. Those zones, shown in Fig. 4, have been denoted by letters, from (a) to (f). The three entry/exit zones are:

- (a) Towards or from the North Atlantic area
- (b) Towards or from the South Atlantic area
- (c) Through the Panama Canal

and the four areas of interest in which our ports of interest are located are:

- (d) the Lesser Antilles
- (e) the Caribbean Sea
- (f) the Gulf of Mexico
- (g) the Atlantic Ocean

For every day of the 2019 year, and for every vessel in the dataset, the presence of that vessel in one or several of those zones, or the absence of the vessel from the zone, is shown in this file.

Table 10 lists all features in this file, given that the feature named “d_XX_YY” accounts for 365 distinct columns, ordered temporally, with XX taking values in [01,12] and representing the month, and YY taking values in [01,31] representing the day. All those 365 columns take values as follow: ‘d’ if the vessel was seen only in the Lesser Antilles area on the day of interest, ‘ce’ if the vessel was seen in both the Panama Canal and the Caribbean Sea on the day of interest, or ‘null’ is the vessel was not present in our dataset for this particular day. This file has 35,652 entries, which is the total number of unique MMSI numbers seen across the year. Please note that all of those vessels do not stop in one of the 528 ports of our dataset (only 22,225 do), and not all of them have their type characteristics known (only 12,476 do, although the missing vessels are mainly of service and pleasure vessels). Table 10 details the characteristics of the fields of this file.

**Table 10:** Description of the data feature of the “areapresence” data file.

Feature	Type	Universe of discourse	Short description
**id**	Integer	[1;35,652]	Vessel internal identifier, primary key
**mmsi**	Integer	[12;1,073,709,206]	Unique vessel identifier number, as defined by the IMO and assigned by countries
**d_XX_YY**	Text	Any combination of {a,b,c,d,e,f,g}	Presence of vessel in any area, or absence thereof

### 3.10 Docking type file

In this file, named docking.csv, three geometries of docking areas, namely all quays, all wharves and all offshore platforms are proposed. They are not differentiated by port but are proposed as a single geometry feature for a differentiated approach of docking sites. Because of the survey method, later presented in Section 4.1.3, redundancies may be present, as well as docking areas that were not, eventually, considered in the port list. Table 11 details the characteristics of the fields of this file.

**Table 11:** Description of the data feature of the “docking” data file.

Feature	Type	Universe of discourse	Short description
**id**	Integer	[1;3]	Unique identifier, primary key
**docktype**	Text	{quay, wharf, offshore}	Type of docking
**geom**	geometry		MultiLineString of docking outlines

### 3.11 Port traffic statistics file

This file, named port_traffic.csv, stands as an example of data aggregation that can be performed using this dataset. It lists all ports, takes port call values and unique vessel values from the **port calls** table, and additionally, provides a range of aggregated data extracted from the IHS Markit fleet register. Two values are indicated: a) the number of ships for which this data is available (which is an important feature for assessing the representativeness of the other values), b) the number of ships for which the age is available. In most cases, both items of information are available or unavailable. One is rarely available if the other is not. The two distinctive values are the total cumulative tonnage of vessels in the given port, and the median age of vessels in the given port. Tonnage is cumulative in the sense that if a vessel visits the port ten times, its tonnage is added ten times as well. Median age is rounded to the nearest integer. Those pieces of information are also available under a disaggregated approach of maritime traffic by vessel type, with the seven vessel types being as previously described in Section 3.7. Table 12 details the characteristics of the fields of this file.

**Table 12:** Description of the data features of the “port traffic” data file.

Feature	Type	Universe of discourse	Short description
**id**	Integer	[1,528]	Unique identifier of the port, references column **portid** of the ports table
**portname**	Text	List of ports	Name of port
**country**	Text	List of territories	Name of territory
**pc_total**	Integer	N+	Total number of port calls
**pc_xxxx**	Integer	N+	7 column, for which xxx stands for {bulk, cont, crui, gcar, iisl, serv, tank}. Total number of port calls for the corresponding vessel type
**uv_total**	Integer	N+	Total number of unique vessels calling at that port
**uv_xxxx**	Integer	N+	7 column, for which xxx stands for {bulk, cont, crui, gcar, iisl, serv, tank}. Total number of unique vessels calling at that port for the corresponding vessel type
**vk_total**	Integer	N+	Number of vessels for which the characteristics (age and probably tonnage) are known
**vk_xxxx**	Integer	N+	7 column, for which xxx stands for {bulk, cont, crui, gcar, iisl, serv, tank}. Number of vessels for which the characteristics (age and probably tonnage) are known for the corresponding vessel type
**tt_total**	Integer	N+	Total cumulative tonnage of vessels calling at that port for which the tonnage is known, in GT
**tt_xxxx**	Integer	N+	7 column, for which xxx stands for {bulk, cont, crui, gcar, iisl, serv, tank}. Total cumulative tonnage of vessels calling at that port for which the tonnage is known, in GT, for the corresponding vessel type
**ma_total**	Integer	N+ or *null*	Median age of vessels calling at that port for which the age is known, rounded to the nearest integer
**ma_xxxx**	Integer	N+ or *null*	7 column, for which xxx stands for {bulk, cont, crui, gcar, iisl, serv, tank}. Median age of vessels calling at that port for which the age is known, rounded to the nearest integer, for the corresponding vessel type

### 3.12 UN country port calls file

This table contains data statistics that have been directly extracted from the United Nations Conference on Trade and Development [UNCTAD]. In this table, for each country or territory, the total number of port calls that have been declared can be retrieved. The ‘abbr’ column is not present in the original UNCTAD, and has been added so that cross-tables queries can be performed. Those data are stored in the file named ungt_country_portcalls.csv and Table 13 lists all features in this file.

**Table 13:** Description of the data features of the “ungt_country_portcalls” data file.

Feature	Type	Universe of discourse	Short description
**country**	Text	Closed List	Short name of the country or territory
**abbr**	Text	Closed List	3-letter country or territory code, according to ISO 3166-1 alpha-3
**allships**	Integer	N	Number of port calls for all vessel types
**liq_bulk**	Integer	N	Number of port calls for vessels of type liquid bulk
**liq_petroleum_gas**	Integer	N	Number of port calls for vessels of type liquid petroleum gas
**liq_natural_gas**	Integer	N	Number of port calls for vessels of type liquid natural gas
**dry_bulk**	Integer	N	Number of port calls for vessels of type dry bulk
**dry_breakbulk**	Integer	N	Number of port calls for vessels of type dry breakbulk
**roro**	Integer	N	Number of port calls for vessels of type roro
**container**	Integer	N	Number of port calls for vessels of type container
**passenger**	Integer	N	Number of port calls for vessels of type passenger

### 3.13 Statistics on UN country port calls file

This table contains data statistics that have been directly extracted from the United Nations Conference on Trade and Development [UNCTAD]. In this table, for each country or territory, and for each vessel type, some characteristics of vessels and port calls are shown, such as the age of such vessels, the median port call time or the gross tonnage of vessels. The ‘abbr’ column is not present in the original UNCTAD, and has been added so that cross-tables queries can be performed. Those data are stored in the file named ungt_country_portstats.csv and Table 14 lists all features in this file.

**Table 14:** Description of the data features of the “ungt_country_portstats” data file.

Feature	Type	Universe of discourse	Short description
**country**	Text	Closed List	Short name of the country or territory
**abbr**	Text	Closed List	3-letter country or territory code, according to ISO 3166-1 alpha-3
**vesseltype**	Text	All vessel type features from Section 3.7	Type of vessel concerned
**med_time**	Real	R+	Median time of port calls for the given country for the given vessel type, in days
**avg_age**	Integer	N	Average age of vessels calling in a port of the given country for the given vessel type, in years
**avg_gt**	Integer	N	Average gross tonnage of vessels calling in a port of the given country for the given vessel type
**max_gt**	Integer	N	Maximal gross tonnage of vessels calling in a port of the given country for the given vessel type
**avg_dwt**	Integer	N	Average deadweight of vessels calling in a port of the given country for the given vessel type
**max_dwt**	Integer	N	Maximal deadweight of vessels calling in a port of the given country for the given vessel type
**avg_teu**	Integer	N	Average capacity of vessels calling in a port of the given country for the given vessel type, in twenty-foot equivalent units
**max_teu**	Integer	N	Maximal capacity of vessels calling in a port of the given country for the given vessel type, in twenty-foot equivalent units

### 3.14 UN container throughput

This table contains data statistics that have been directly extracted from the United Nations Conference on Trade and Development [3]. In this table, for each country or territory, the total (cumulative) amount of the capacity of all vessels calling in the country, in twenty-foot equivalent units. The ‘abbr’ column is not present in the original UNCTAD, and has been added so that cross-tables queries can be performed. Those data are stored in the file named ungt_cont_throughput.csv and Table 15 lists all features in this file.

**Table 15:** Description of the data features of the “ungt_cont_throughput” data file.

Feature	Type	Universe of discourse	Short description
**country**	Text	Closed List	Short name of the country or territory
**abbr**	Text	Closed List	3-letter country or territory code, according to ISO 3166-1 alpha-3
**teu**	Integer	N	Cumulative capacity of vessels, in teu, for the corresponding country

## 4 Experimental Design, Materials and Methods

**Fig. 5:**
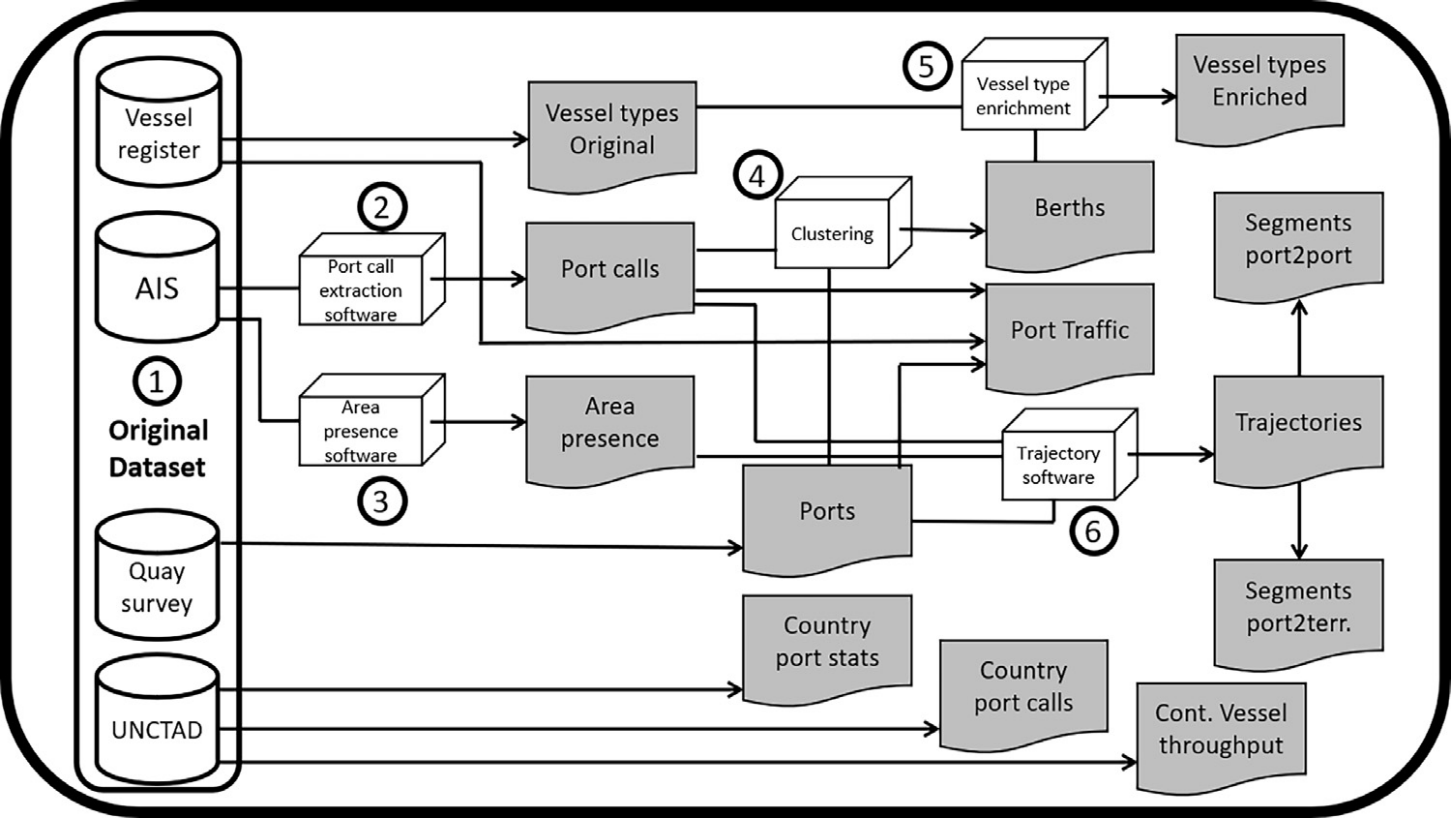
Workflow for dataset generation.

Fig. 5 presents a synoptic schematic representation of the methods used for the generation of the dataset. The different parts will be further detailed in this section. On top of descriptive features, a geovisualisation dashboard that allows navigation in this very dataset has been developed and is available online [6].

### 4.1 Original dataset

In this section, the data sources for crafting the dataset that this article describes are presented. The raw AIS original dataset (described in Section 4.1.1) and the vessel registry dataset (described in Section 4.1.2) are not part of our release. They have been both purchased from commercial companies, and the data we share in the eleven original tables of our dataset have been carefully filtered, selected, crafted and transformed from our own original algorithms. They resemble in no way to the original purchased data, and the publication of this dataset is in accordance with licence and data ownership rights.

#### 4.1.1 AIS dataset

The dataset detailing vessel positions was purchased from the company exactEarth [1] and covers the Caribbean Sea, the Gulf of Mexico, and surrounding areas. Spatially, the data ranges from 3.86 to 34.05 degrees North latitude and 98.02 to 51.17 degrees West longitude. The dataset is extensive, featuring a cumulative total of 641,709,724 data contacts, averaging about 1.76 million AIS messages daily. This large volume necessitates cautious processing to ensure manageable computation times. The use of AIS data is common when measuring the activities of vessels at sea [7] and offers a good picture of the maritime situation, despite some issues with the system [8]. Buying data from a provider is a common practice, and researches using exactEarth company data can be found [9,10]

**Fig. 6:**
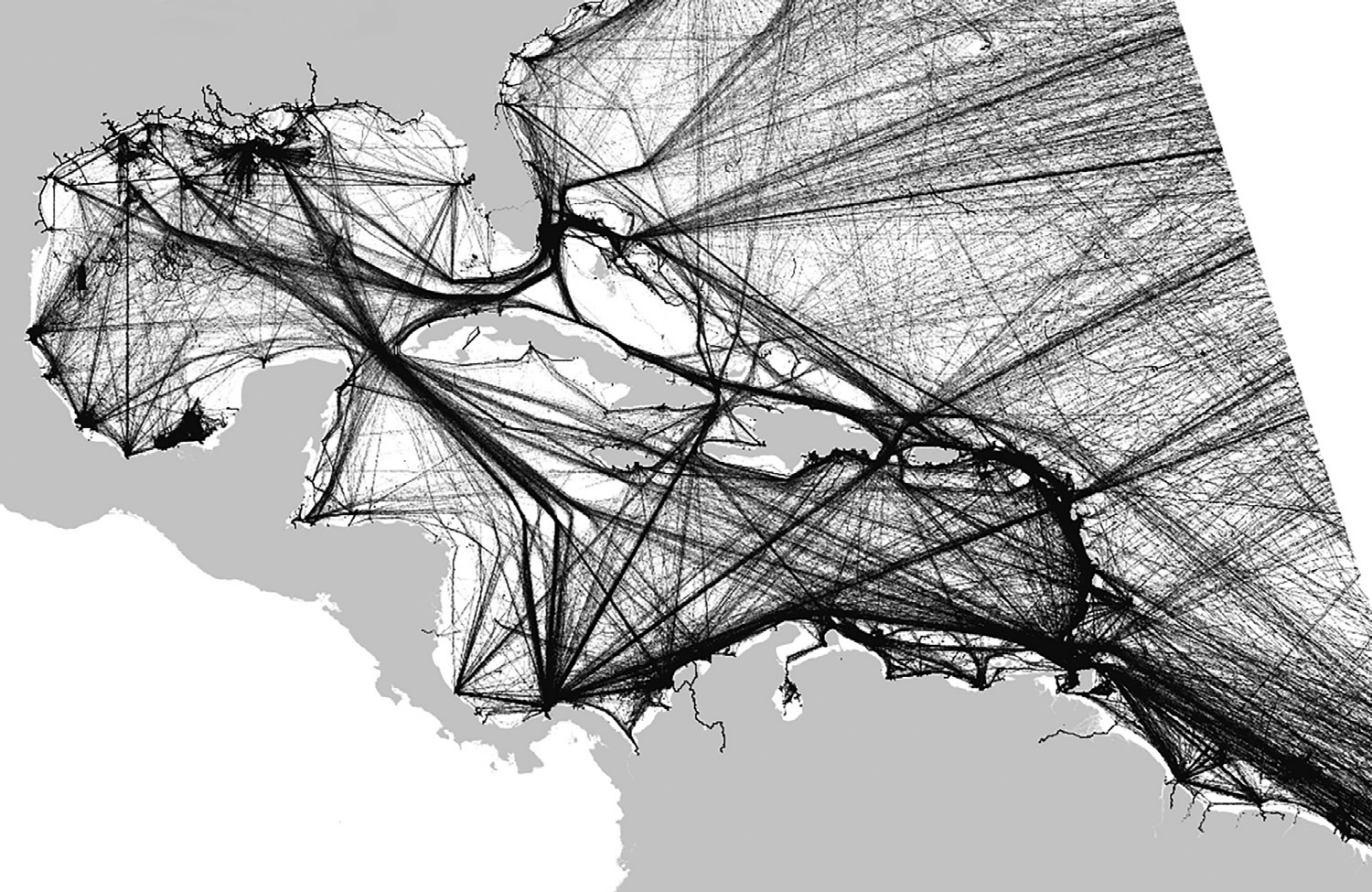
The original AIS dataset.

Fig. 6 presents data contacts for January 2019 alone, which includes 46 million messages, and shows the spatial bounds of our dataset. The dataset follows the message formats specified by the International Telecommunication Union, namely messages number 1, 2, 3, 18, 19, and 27. It spans a time period from January 1 to December 31, 2019, and employs the WGS84 coordinate system for location data.

The messages, captured via satellite, include various key parameters such as the Maritime Mobile Service Identity (MMSI), coordinates, speed over ground in knots, true heading, and course over ground relative to True North, as well as the rate of turn in degrees per minute and the current navigational status of the vessel. Since AIS messages lack an emission timestamp, a reception timestamp is appended during data parsing.

#### 4.1.2 Vessel register

To achieve a nuanced understanding of vessel differentiation, we employed a type-based categorization, leveraging the classification system of the IHS Markit [2] database, that we purchased. The categories under investigation include bulk carriers, container ships, cruise vessels, general cargo vessels, inter-island ships, service vessels, and tankers. It should be noted that our focus is restricted to commercial fleets and service vessels, deliberately excluding fishing and recreational vessels.

However, the classification schema is not without limitations. Certain vessels fitting one of the selected categories may either be absent from the IHS Markit database or classified differently. Additionally, the IHS Markit categories possess varying degrees of granularity, necessitating data retrieval from multiple fields and thereby increasing the risk of misclassification. Nonetheless, to the best of our knowledge, these categories are mutually exclusive.

#### 4.1.3 Quay survey

While global databases of ports do exist, they tend to focus on major ports and often lack both comprehensiveness and accuracy [11]. For instance, the World Port Index (WPI) lists 280 Caribbean ports, and the IHS Markit database accounts for 301, compared to the 528 commercial port sites identified in our research. The spatial information for these ports is often approximate, represented merely by a point, without spatialized representation of berthing infrastructures and terminals. To establish a more accurate cartographic frame of reference for port call calculations, we have created a dataset describing the infrastructures, including quays and wharves, for all Caribbean port sites identified by photo-interpretation. The entire coastline of the greater Caribbean region was explored at a scale of 1:5000, mainly using *Google Satellite* and *Bing Maps*. As our research focused on commercial shipping, port sites dedicated exclusively to pleasure boating were not digitised. To identify passenger embarkation and disembarkation areas, we used *Google Earth* and the *Open Street Maps* cartographic repository.

Our differentiation of berthing structures was designed as follows: a wharf is a platform on stilts along which a ship docks; offshore are berthing structure not connected to land; quays are all other berthing structures.

Using aerial photos or satellite images taken vertically, it is not always easy to see the difference between quays and wharves. To limit some of the bias, a second photo-interpreter re-explored the entire dataset to standardise interpretation.

#### 4.1.4 Reference data from UNCTAD

Data sourced from UNCTAD (United Nations Conference on Trade and Development) is abundant but is aggregated at the level of countries or dependencies, rather than individual ports. To evaluate our port call computations, we used a table extracted from the UNCTAD website [3], which we subsequently restructured within our own database. This modified table delineates the annual number of port calls for various vessel types—namely, liquid bulk, dry bulk, dry breakbulk, liquefied petroleum gas, liquefied natural gas, roll-on roll-off vessels, container ships, and passenger vessels—across different territories for the year 2019.

### 4.2 Port call extraction software

In this section, we describe the method used for the generation of the set of port calls, which has been tested and validated as shown in [5].

**Fig. 7:**
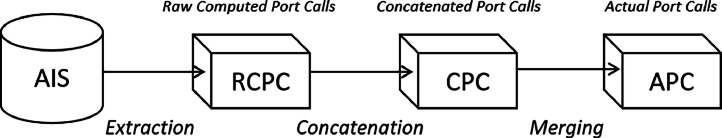
Schematic succession of operations leading to the published port calls.

The computation of port calls is performed in three steps, as shown in Fig. 7. First, AIS data is retrieved and the string of data points that display the behaviour of a port call are individuated into Raw Computed Port Calls (RCPCs), for which characteristics such as the length and the location are computed. Then, two consecutive operations, namely the concatenation and the merge, are performed, generating the set of Actual Port Calls (APCs) that we will use for the remainder of this article. The first step is shown in Section 4.2.1 and both concatenation and merge operations are shown in Section 4.2.2.

#### 4.2.1 Computation of raw calls

The first step of the generation of the port calls dataset is the computation of raw port calls.

**Fig. 8:**
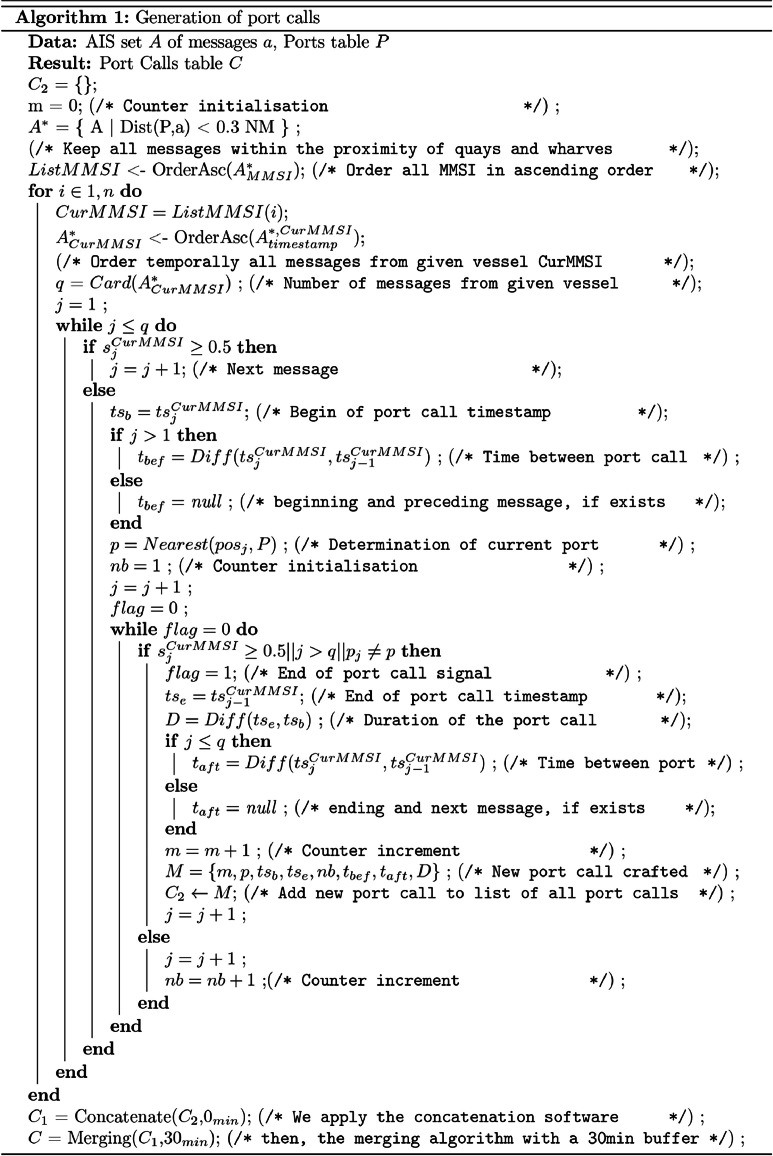
Algorithm describing the extraction of port call from raw AIS data and additional data sets.

Raw computed port calls (RCPC) are generated as follows: we consider all AIS messages sent with a recorded position within a buffer of ca. 600m around the surveyed quays and wharves. Out of this reduced dataset, each vessel trajectory is considered separately and, in succession, all cases for which the speed over ground of a vessel goes below the speed threshold of 0.5kn then, after some time (very short or very long), provided that the vessel has remained in the vicinity of the same port, its speed over ground goes up, crossing again the speed threshold of 0.5kn, constitute a raw port call [12]. Fig. 8 shows the algorithmic procedure for the computation of those port calls.

#### 4.2.2 Artefact removal using concatenation and merging

To produce the final table encompassing all ports of call, two distinct and sequential processes are employed. Initially, a series of Raw Consecutive Port Calls (RCPCs) are concatenated, forming what are termed Concatenated Port Calls (CPCs). Following this, the CPCs undergo a merging process if the interval separating them is sufficiently short. This two-step procedure ensures the comprehensive and accurate representation of port call data in the final table.

The concatenation process arises from the computational approach used. Specifically, Raw Consecutive Port Calls (RCPCs) are calculated on a per-vessel basis. This is due to the fact that vessels remaining in the port area for extended periods tend to transmit a substantial volume of messages. To circumvent the need to manage excessively large data arrays and to conserve computational resources, the data from each individual vessel is segmented into several data blocks. The number of these blocks correlates directly with the total count of messages received from that particular vessel. This method streamlines data processing, making it more manageable and time-efficient.

Hence, in instances where a data block terminates amidst a port call, the subsequent data block commences during the same port call. This results in the computation identifying two distinct Raw Consecutive Port Calls (RCPCs). Moreover, a sufficiently lengthy port call might extend across more than two data blocks. Our computational workflow is designed to retrieve the first message preceding and following each RCPC. Utilising these timestamps enables the merging of these RCPCs. Consequently, we can recalculate the attributes of the newly formed Concatenated Port Call (CPC), reflecting the actual duration and characteristics of the port call as it occurred. This process involves synthesising the attributes of each individual RCPC that constitutes the CPC.

**Fig. 9:**
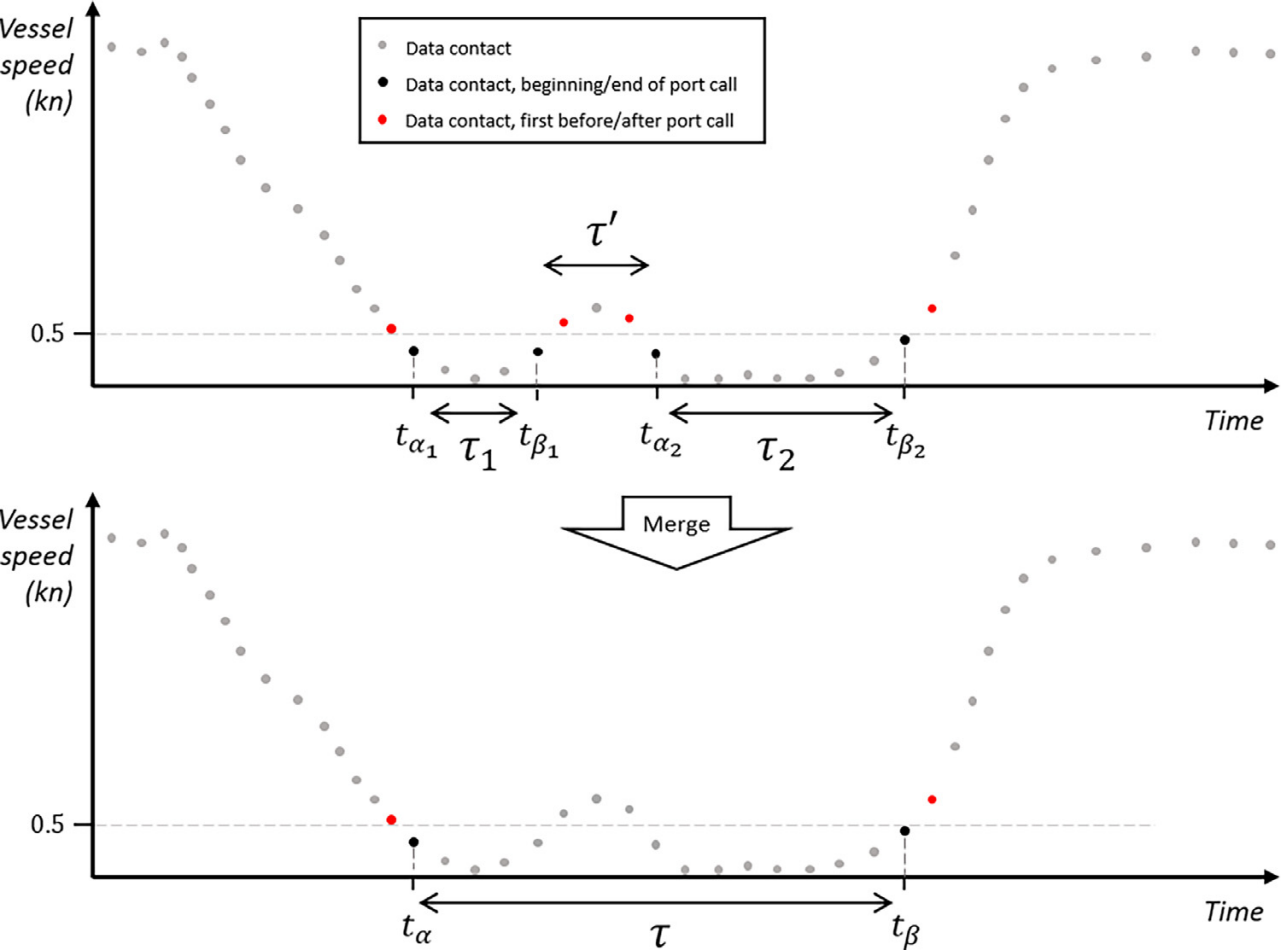
Schematic representation of the merging of two consecutive CPCs into a single APC.

The merging of Concatenated Port Calls (CPCs) takes place when two such calls for the same vessel at the same port occur consecutively. This process is depicted in Fig. 9. Between these CPCs, there is an interval where the vessel achieves a speed exceeding a predetermined threshold, effectively concluding one CPC computationally. Various factors can contribute to this interlude between two CPCs: minor vessel relocations due to currents, transitioning from one dock to another, brief movements following arrival, or GPS inaccuracies leading to perceived movement, among other reasons.

It's important to note that this merging is not limited to just two CPCs; it can involve multiple consecutive CPCs for the same vessel at the same port. During such occurrences, the computation process consolidates these into a single new Actual Port Call (APC), with all the original CPCs being replaced by this aggregated entity. The characteristics of the port call are then recalculated based on this new APC. For clarity and ease of explanation, Fig. 9 shows and focuses on the scenario where only two CPCs are involved in the merging process. Number of calls in each computation step and in each geographical area are shown in Table 16.

**Table 16:** Number of computed port calls at each step of the computation for the five geographical areas of interest.

	RCPC	CPC	APC
Lesser Antilles (101 ports)	375,387	374,692	171,262
Caribbean Sea (176 ports)	443,985	443,073	206,675
Gulf of Mexico (63 ports)	1,922,512	1,920,035	934,416
Outer areas (47 ports)	228,505	228,023	142,167
Minor ports (141 ports)	71,977	71,886	38,745
Total	3,042,366	3,037,709	1,493,265

#### 4.2.3 Confidence coefficient for port call duration

Apart from the determination of the occurrence of a port call, another variable of interest is the duration of the call, as this duration may indicate various levels of activity in various ports, particularly when this duration is compared against other ports for similar vessels.

In the context of processing AIS data, accurately calculating the duration of a port call presents challenges due to the dependency on the quality of the available data. Although it's feasible to determine whether a port call occurred despite data gaps, assigning an accurate duration to that call requires certain criteria to be fulfilled: both arrival and departure times need to be clearly established, and the time series should not exhibit significant temporal discontinuities. Consequently, it becomes crucial to compute a confidence coefficient. This metric allows for the evaluation of the reliability of the calculated times, ensuring they are treated appropriately in light of their quality.

**Fig. 10:**
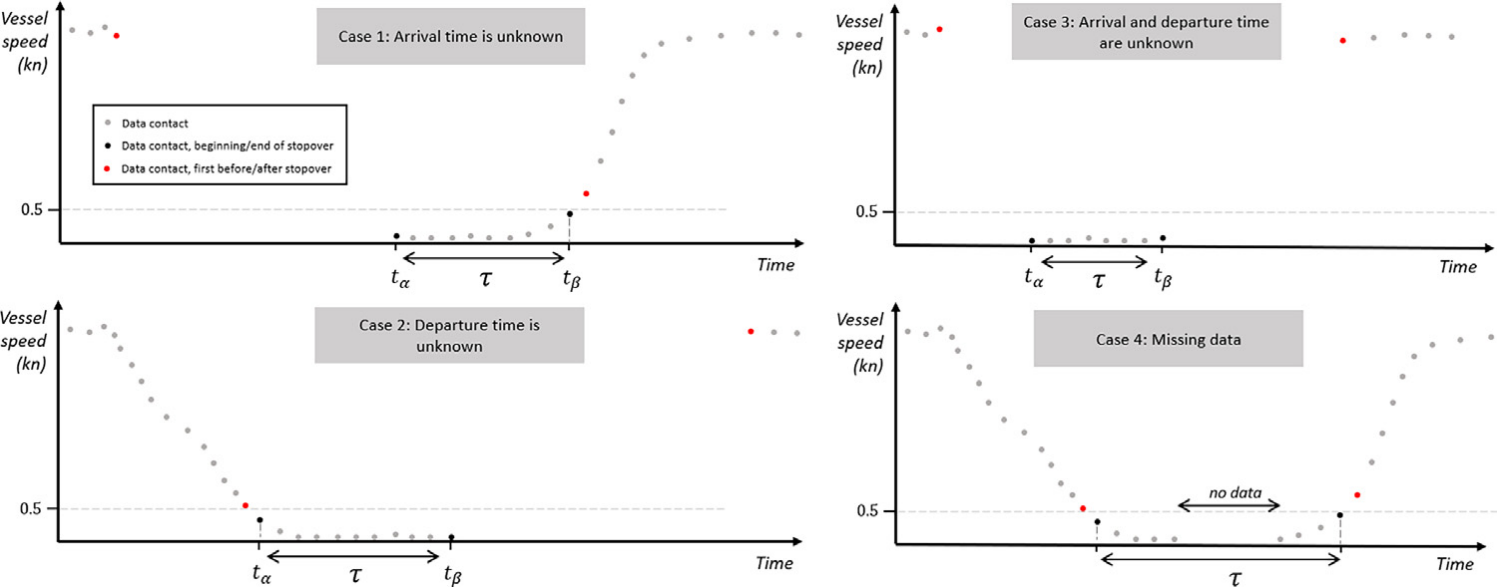
Schematic representations of the four cases in which the duration of a port call cannot be established with the highest certainty.

Fig. 10 illustrates four scenarios where it's evident that a port call occurred, yet any temporal assessment of its duration remains uncertain. In Case 1, the vessel's arrival data is missing, leading to an underestimation of the actual port call duration. Case 2 mirrors this situation, with the departure data missing instead, again resulting in an underestimated port call duration. Case 3 combines the issues of Cases 1 and 2: data is absent for both the vessel's arrival and departure, likely causing a significant underestimation of the port call duration. Case 4 presents a different challenge; while arrival and departure data are available, there is a notable gap in reporting during the port call. Consequently, while the calculated duration may be accurate, it's uncertain if the vessel remained stationary throughout this period. There's a possibility that what is recorded as a single port call could, in fact, be two separate calls of varying lengths.

The computation of the coefficient uses the following parameters: the time between the first message within the port call and the last message received beforehand, shown in red in Fig. 10, the time between the last message within the port call and the first message afterwards, shown in red in Fig. 10, and a factor denoting the degree to which no major gap between messages can be stopped in the timeframe of the port call. A given time above which the duration is deemed as too long for both times has been set at six hours.

### 4.3 Area presence software

The Area Presence Software is an integral component of our study, tasked with determining vessel locations within the Caribbean region. This region, our primary area of interest, requires precise monitoring of maritime traffic. The software analyses position messages transmitted by vessels, which contain positional data. To facilitate this analysis, the Caribbean is segmented into seven specific zones (cf. Section 3.9). Four of these are central areas: the Gulf of Mexico, the Lesser Antilles, the Caribbean Sea, and the Atlantic Ocean. The remaining three are strategic entry/exit areas: the North Atlantic, South Atlantic, and the Panama Canal. This segmentation is essential for understanding vessel movements, particularly when tracking their entry into or exit from the Caribbean.

The methodology for data processing involves organising vessel position data into daily batches. Each day, the software systematically processes each position message from every vessel. Through spatial intersection analysis, it determines whether a vessel is within the Caribbean's boundaries and, subsequently, identifies the specific area (or, if applicable, areas) it occupies. This step is crucial for understanding the daily distribution and movement of maritime traffic within the region.

To track and record vessel movements effectively, each of the seven areas is assigned a unique letter identifier. This aspect of the software design takes into account that vessels can move across various areas in a single day. To accurately reflect these movements, the software concatenates the letters corresponding to each area a vessel travels through during the day. The resulting string, representing the vessel's trajectory, is then inserted into our database. This method not only simplifies the representation of vessel movements but also enhances the granularity of our data analysis. The various areas are concatenated by alphabetical order and do not reflect the order in which the areas have been crossed.

This piece of software enables the detailed monitoring of vessel movements within the Caribbean, and by pinpointing when vessels enter or exit the Caribbean and tracking their intra-regional movements, the software provides a comprehensive view of maritime traffic patterns. This capability is fundamental to our analysis, offering insights into the dynamics of maritime traffic in this strategically important area.

### 4.4 Clustering

In our study, a critical step involved generating a comprehensive “berths” table that captures the frequent stopping locations of vessels during their port calls. This table builds upon an existing table of maritime port calls, which was previously calculated (see Section 4.2 and Section 3.2), as well as the geometric layout of quays and docks within ports (see Section 3.3).

The purpose of creating the “berths” table was to identify and catalogue clusters of frequent berthing locations, represented as unique polygonal geometries. This would provide a more nuanced understanding of port activity, allowing for specific locational analysis within a port, beyond general port calls characteristics.

The clustering was manually performed using the *QGIS* piece of software. This manual approach was necessary to ensure the utmost precision and to accommodate the unique geometric configurations of various quays and wharves. It also permitted greater control in resolving ambiguities that could arise from a purely algorithmic approach, such as overlapping polygons or adjacent berthing locations that needed to be treated as separate entities.

Each entry in the “berths” table was systematically named based on the identification number of the port in question. This not only ensures uniformity in the data but also facilitates easier cross-referencing with the original table of port calls and other related datasets.

In summary, it represents a meticulous manual effort to capture the intricacies of berthing locations within ports, thereby enriching our understanding of maritime logistics and activities at a fine granular level.

### 4.5 Vessel type enrichment

To enhance our dataset with vessel types, we generated a new table called “vesseltype_enriched”. The objective of this table is to gather vessels for which the type could be inferred. The basis for this inference came from two primary sources: first, a commercially acquired vessel register from IHS Markit, for which vessels are stored in the table “vesseltype_original”, and second, our previously generated “berths” table.

The underlying hypothesis for this enrichment strategy is that vessels of the same type are likely to dock at the same berths. Using this assumption, we cross-referenced the known types from the IHS Markit register with the berths where vessels frequently dock, as indicated in our “berths” table. This allowed us to infer the types of vessels that were previously unidentified.

Following the inference step, we implemented stringent validation measures. Only the vessels for which at least 90% of the known port calls at shared berths were from a single, identified vessel type were retained. Furthermore, this 90% category needed to make up at least half of all known port calls for a particular berth to be deemed reliable. These validation criteria were established to ensure the robustness and accuracy of the “vesseltype_enriched” table. This method enabled us to enrich the vessel information of 2,643 vessels with their inferred type, using data from 1,902 berths and 12,476 vessels of previously known type.

### 4.6 Trajectory software

The generation of the vessel trajectory data table necessitated a dedicated software approach that integrated data from multiple pre-existing tables to build detailed and chronologically accurate maritime paths.

The primary foundational information for the location and timestamp of vessel stops was sourced from the **Port Calls** table. This provided a chronological ordering to the journey, making it a reference for the entire trajectory reconstruction process. Adding a layer of precision to this, the **Area Presence** table recorded days when vessels were located at the limits of the Caribbean zone. This was essential to ensure trajectory accuracy, as it allowed for the identification of when vessels might be transitioning in or out of the region. Hence, it acted as a mechanism to prevent potential inaccuracies that could arise, such as mistakenly designating two port calls as sequential when they might actually be separated by the vessel exiting the Caribbean and then returning at a later point. In this respect, specific numbers were assigned to the three entry/exit areas, as shown in Section 3.4. Lastly, the **Ports** table enriched the trajectory determination with data about the ports, granting further context to the port calls and allowing to build geometries based on the location of the quays.

Trajectories of vessels were processed individually. Each vessel, identified by its MMSI number, was taken up in ascending order for methodical processing. For every vessel, the associated port calls were organised chronologically. Intercalated within this sequence were the recorded days from the **Area Presence** table, which indicated the vessel's proximity to the edges of the Caribbean zone.

**Fig. 11:**
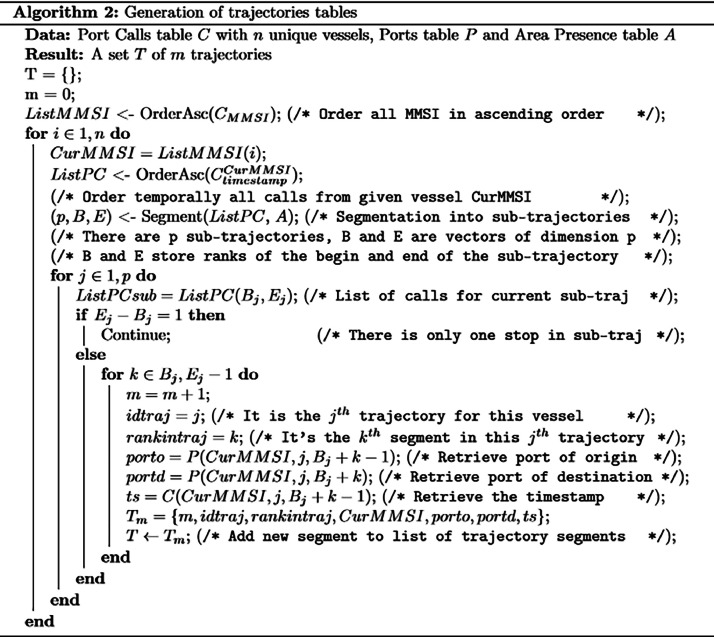
Algorithm for the generation of the “trajectories” table.

Every segmented trajectory, thus identified, was logged into the database. To maintain clarity and aid in detailed analyses, every journey segment within each trajectory was uniquely numbered. This meticulous approach, rooted in the synthesis of multiple data sources, resulted in the creation of a comprehensive vessel trajectory table that not only detailed individual voyages, but also shaped the broader maritime movement patterns within the Caribbean region. Fig. 11 shows the algorithm for the generation of this table.

## Limitations

The port calls dataset has been computed from raw AIS data that has their own intrinsic limitations. Although the vast majority of the data faithfully represents the activity of seagoing vessels, data may be marginally missing or display erroneous features as some elements, such as the vessel identity number (MMSI) is input manually. For instance, in this dataset, 0.25% of all computed port calls display an incorrect MMSI number.

This dataset is also geographically bounded by the area for which raw AIS data is available in our study. However, the proposed method can be applied anywhere, provided that raw AIS data is available, and the algorithms showed in the corresponding section are not area-specific.

## Ethics Statement

This dataset has been crafted from raw AIS data purchased from exactEarth and raw vessel information data purchased from IHS Markit. It has undergone extensive curation, cleaning, and processing, significantly transforming it from its original form. The data originally acquired for this study were subject to rigorous refinement procedures to ensure accuracy, relevance, and utility in our analysis. It is important to emphasise that the data in their current form, as presented in this paper, bear no resemblance to the raw data initially collected. This transformation was conducted with the utmost ethical considerations, maintaining the integrity of the data while ensuring that it meets the high standards required for scientific research and analysis.

## CRediT authorship contribution statement

**Clément Iphar:** Conceptualization, Data curation, Funding acquisition, Methodology, Software, Visualization, Writing – original draft. **Iwan Le Berre:** Conceptualization, Data curation, Funding acquisition, Supervision, Writing – review & editing. **Manuel Sahuquet:** Conceptualization, Data curation, Writing – review & editing. **Aldo Napoli:** Funding acquisition, Supervision, Writing – review & editing. **Éric Foulquier:** Funding acquisition, Supervision, Writing – review & editing.

## Data Availability

Port calls and vessel trajectory dataset in the Caribbean with accurate port quays survey (Original data) (Zenodo).
